# Structural and biophysical analysis of nuclease protein antibiotics

**DOI:** 10.1042/BCJ20160544

**Published:** 2016-09-12

**Authors:** Alexander Klein, Justyna Aleksandra Wojdyla, Amar Joshi, Inokentijs Josts, Laura C. McCaughey, Nicholas G. Housden, Renata Kaminska, Olwyn Byron, Daniel Walker, Colin Kleanthous

**Affiliations:** 1 Department of Biochemistry, University of Oxford, South Parks Road, Oxford OX1 3QU, U.K.; 2 Swiss Light Source, Paul Scherrer Institut, 5232 Villigen PSI, Switzerland; 3 Institute of Infection, Immunity and Inflammation, College of Medical, Veterinary and Life Sciences, University of Glasgow, Sir Graeme Davies Building, 120 University Place, Glasgow G12 8TA, U.K.; 4 School of Life Sciences, University of Glasgow, University Place, Glasgow G12 8TA, U.K.

**Keywords:** bacteriocin, colicin, crystallography, pyocin, small-angle scattering

## Abstract

Protein antibiotics (bacteriocins) are a large and diverse family of multidomain toxins that kill specific Gram-negative bacteria during intraspecies competition for resources. Our understanding of the mechanism of import of such potent toxins has increased significantly in recent years, especially with the reporting of several structures of bacteriocin domains. Less well understood is the structural biochemistry of intact bacteriocins and how these compare across bacterial species. Here, we focus on endonuclease (DNase) bacteriocins that target the genomes of *Escherichia coli* and *Pseudomonas aeruginosa*, known as E-type colicins and S-type pyocins, respectively, bound to their specific immunity (Im) proteins. First, we report the 3.2 Å structure of the DNase colicin ColE9 in complex with its ultra-high affinity Im protein, Im9. In contrast with Im3, which when bound to the ribonuclease domain of the homologous colicin ColE3 makes contact with the translocation (T) domain of the toxin, we find that Im9 makes no such contact and only interactions with the ColE9 cytotoxic domain are observed. Second, we report small-angle X-ray scattering data for two S-type DNase pyocins, S2 and AP41, into which are fitted recently determined X-ray structures for isolated domains. We find that DNase pyocins and colicins are both highly elongated molecules, even though the order of their constituent domains differs. We discuss the implications of these architectural similarities and differences in the context of the translocation mechanism of protein antibiotics through the cell envelope of Gram-negative bacteria.

## Introduction

The three-layered structure of the Gram-negative cell envelope, comprising the outer membrane, inner membrane, and intervening peptidoglycan, is a formidable defensive barrier. Nevertheless, antimicrobial peptides and proteins from competing bacteria can breach these defences. Such competition systems are an integral part of the cohabitation of bacteria in mammalian hosts and are often associated with pathogenesis [1]. Three forms of protein-mediated bacterial antagonism are known to exist: bacteriocins (protein antibiotics), which are released into the extracellular environment, contact-dependent inhibition (CDI), and type VI secretion [2–4]. All three forms of antagonism result in death of a targeted cell by a toxin to which the producing strain is usually resistant. Many similarities are beginning to emerge between the modes of action of bacteriocins, CDIs, and the type VI secretion system, most notably in the types of cytotoxic activities delivered to the susceptible cell.

The present work centres on species-specific protein antibiotics that target Gram-negative bacteria, colicins that kill *Escherichia coli*, and pyocins that kill *Pseudomonas aeruginosa* [5,6]. The species specificity of these and related toxins arises from many protein–protein interactions involved in their uptake. Colicins bind to cell surface receptors, usually nutrient transporters, porins, or surface glycolipids, and then contact proton motive force (pmf)-linked protein machines within the periplasm and inner membrane (Tol-Pal or Ton) via outer membrane translocator proteins in order to promote their import [7,8]. E-type colicins parasitize the vitamin B_12_ receptor, BtuB, as their primary receptor in the outer membrane [9] and the Tol-Pal complex in the periplasm [5]. S-type pyocins S2 and S5 bind FpvA and FptA, respectively, which import siderophore-chelated iron [10,11]. How they translocate across the outer membrane is not known, although pyocin AP41, the receptor of which has yet to be identified, may translocate into cells via the Tol-Pal system [12].

Colicin-induced cell death ensues through one of several mechanisms: an ionophore that depolarizes the inner membrane, a periplasmic enzyme that cleaves lipid-linked peptidoglycan precursors, or a nuclease that cleaves nucleic acid substrates in the cytoplasm (DNA, tRNA, or rRNA) [5]. Similar to colicins, S-type pyocins exhibit the same spectrum of cell-killing activities; pyocins S1–S3 and AP41 are DNases, pyocin S4 is a tRNA-specific RNase (tRNase), pyocin M is a peptidoglycan hydrolase, and pyocin S5 is an ionophore [6]. Here, we focus on DNase colicins and pyocins, which, although specific for *E. coli* and *P. aeruginosa*, respectively, deliver a structurally and mechanistically equivalent nuclease to the cytoplasm of each organism to elicit cell death through random cleavage of chromosomal DNA. The catalytic centre of most colicin and pyocin DNases is the HNH/ββα-Me motif, a 34-amino acid motif containing invariant histidine residues, which chelates a single divalent metal ion that catalyzes phosphodiester bond hydrolysis [13]. All colicinogenic and pyocinogenic bacteria also produce an immunity (Im) protein to protect the organism from its own toxin and, in the case of colicins, a bacteriocin release protein or lysis protein that releases the toxin complex to the environment [14]. DNase-specific Im proteins are small acidic inhibitors that bind to an exosite and inhibit colicin or pyocin activity indirectly by blocking substrate-binding [15]. Im proteins are known to dissociate from colicins at the cell surface in a pmf-dependent step by a process that is thought to involve structural remodeling of the nuclease [16,17].

Colicins come in a variety of shapes and sizes (30–80 kDa) [7]. Notwithstanding this diversity colicins invariably have a similar domain arrangement, where a central receptor-binding (R) domain is flanked by an N-terminal translocation (T) domain and a C-terminal cytotoxic (C) domain. Much less is known about the domain arrangement of pyocins although they also have cytotoxic domains located at the C-terminus, while an N-terminal domain is responsible for receptor-binding [18–20]. We report the structure for the 61.5 kDa ColE9 protein in complex with Im9, only the second intact nuclease colicin structure to be determined, and biochemical and biophysical data that probe the domain architecture of DNase pyocins S2 (73.8 kDa) and AP41 (83.9 kDa) in complex with their respective Im proteins.

## Results and discussion

### Structure of the ColE9–Im9 complex and its comparison with other nuclease colicin structures

The only reported structure for a nuclease colicin with all three of its structured domains (T, R, and C) is the ribosomal ribonuclease (rRNase) colicin, colicin E3 (ColE3) [21]. Colicin E9 (ColE9) is ∼90% identical with ColE3 in its R- and T-domains, but differs in having a C-terminal DNase domain and consequently a different Im protein. Notwithstanding the high degree of sequence identity between them (indeed between most E-group colicins), previous kinetic and thermodynamic data have shown that ColE3 and ColE9 differ with respect to how they interact with their specific Im proteins. Both bind their Im proteins with very high affinity (*K*_d_ ∼ 10^−14^ M at pH 7 and 25°C). However, the isolated rRNase of ColE3 binds Im3 more weakly (*K*_d_ ∼ 10^−12^ M at pH 7 and 25°C) than does the DNase of ColE9 where no difference in affinity is observed for Im9 binding the colicin and isolated DNase [22,23]. These observations are consistent with ColE3 making additional interactions with Im3 outside of the rRNase domain, which are predicted to be absent from ColE9. To probe this difference, we determined the structure of intact ColE9 (582 amino acids) in complex with Im9 (86 amino acids).

**Figure 1.**
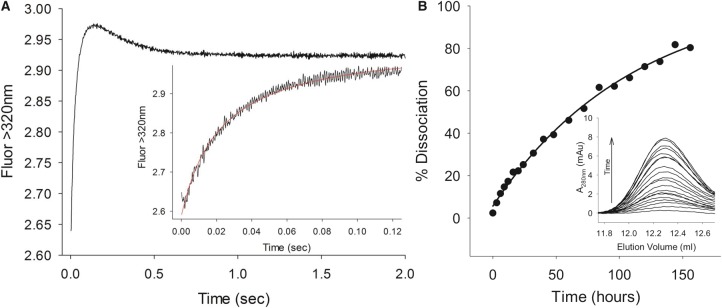
Measurement of ColE9–Im9 association and dissociation kinetics. (**A**) Association kinetics of Y324C, L447C ColE9–Im9 complex monitored through tryptophan fluorescence (*λ*_ex_ = 280 nm and *λ*_em_ > 320 nm) under second-order conditions in PBS, pH 7.4. Equimolar concentrations of the two proteins (0.7 µM before mixing) were mixed at 25 °C; the initial fluorescence enhancement and subsequent quench in fluorescence were fitted to second-order and first-order rate equations, respectively. Complex formation between Y324C, L447C ColE9 and Im9 is characterized by a bimolecular collision with a rate constant (*k*_1_) of 1.13 ± 0.06 × 10^8^ M^−1^ s^−1^, followed by a conformational change with an apparent rate constant (*k*_2_ app) of 2.49 ± 0.02 s^−1^ (values derived from three independent measurements). These values are comparable to those we observed for wild-type ColE9 under the same conditions (*k*_1_ = 1.28 ± 0.03 × 10^8^ M^−1^ s^−1^ and *k*_2_ app = 2.76 ± 0.02 s^−1^; data not shown) and very similar to those reported previously [22]. (**B**) Dissociation of Im9 from the Y324C, L447C ColE9–Im9 complex monitored through the accumulation of E9 DNase–Im9 complex as a function of time. Inset: Quantification of E9 DNase–Im9 complex through size-exclusion chromatography on a Superdex 200 10/300 GL column. The dissociation rate constant of 2.5 ± 0.2 × 10^−6^ s^−1^ determined here, in duplicate for Y324C, L447C ColE9–Im9 in PBS, pH 7.4, is in good agreement with that determined previously for wild-type ColE9 (2.1 × 10^−6^ s^−1^ in 50 mM Mops, pH 7.0, 200 mM NaCl) [22].

Extensive crystallization experiments of the ColE9–Im9 complex were unsuccessful. We rationalized that this might be due to flexibility within the coiled-coil R-domain, which undergoes conformational changes during colicin entry into cells [24,25]. We therefore switched to using a disulphided form of ColE9 in which Tyr324 and Leu447 in the R-domain were mutated to cysteine, which has been reported previously. A disulphide bond forms spontaneously between these mutated residues, that does not influence binding of the toxin to the colicin's receptor BtuB, and has been used to isolate the intact colicin outer membrane translocon complex [26]. However, it has never been ascertained whether the disulphide bond affects the affinity of the ColE9–Im9 complex. We therefore determined the association and dissociation rate constants for the disulphided form of the complex and compared these values to the wild-type complex (Figure 1). Both rate constants (and hence the kinetically determined *K*_d_ ∼ 10^−14^ M) were very similar to those of the wild-type ColE9–Im9 complex reported previously [22], indicating that the disulphide bond has no impact on Im9 binding.

**Figure 2.**
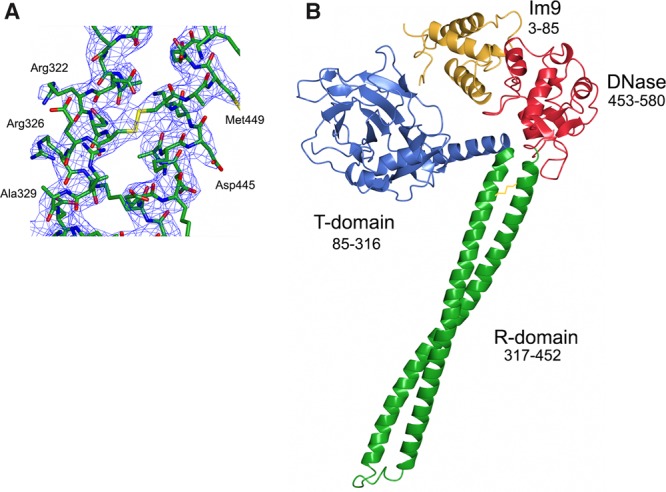
Crystal structure of ColE9. (**A**) Electron density (2*F*_o_ − *F*_c_ shown at 1*σ* cut-off) of R-domain region with stabilizing disulphide bond. (**B**) Structure of the ColE9–Im9 complex shown in cartoon representation with each of the colicin domains labelled. ColE9 T-domain coloured blue, R-domain coloured green, DNase domain coloured red, and Im9 coloured yellow. No electron density for ColE9 residues 1–84 was observed, which is a region known from NMR spectroscopy to be intrinsically disordered [27].

We obtained X-ray diffracting crystals of this disulphide locked form of the ColE9–Im9 complex in the *P*2_1_ space group, and the structure was refined to a resolution of 3.2 Å (see Table 1 and Figure 2A). The asymmetric unit contains four ColE9–Im9 complexes, two pairs of molecules related by non-crystallographic two-fold symmetry. Of these four complexes, two are more rigid with lower B factors and better quality of electron density maps, most probably due to more extended crystal contacts with symmetry-related molecules. Nevertheless, the position of Im9, relative to the colicin, is the same in all four complexes and does not appear to be affected by crystal contacts (Supplementary Figure S1). In the following analysis, the complex with the lowest B-factors for both Im protein and colicin (chains A and E) was chosen for further analysis. The final model of the ColE9–Im9 complex contains 488 residues of ColE9 (85–580) and 83 residues of Im9 (3–85; Figure 2B). No electron density was visible for the first 84 N-terminal residues of ColE9 that comprise the intrinsically unstructured translocation domain (IUTD) of the colicin, consistent with nuclear magnetic resonance spectroscopy data showing that this domain is disordered [27]. Similarly, no density was observed for the IUTD in the crystal structures of ColE3 or the isolated T-domain of ColE7 [21,28]. Clear density was observed for the disulphide bond that had been engineered in the coiled-coil region of the ColE9 R-domain (Figure 2A).

**Table 1:** Data collection and refinement statistics

Data collection	
Space group	*P*12_1_1
Cell dimensions (Å, °)	*a* = 94.9, *b* = 116.1, *c* = 150.4
	*α* = 90.0, *β* = 93.7, *γ* = 90.0
Wavelength (Å)	0.9795
Resolution range (Å)	29.6–3.2 (3.3–3.2)*
Mean *I*/*σ*(*I*)	7.7 (1.8)*
*R*_sym_^†^ (linear) (%)	12.7 (69.4)*
Multiplicity	3.5 (3.4)*
Total reflections	187 563 (14 964)*
Unique reflections	53 941 (4442)*
Completeness (%)	99.1 (95.7)*
Refinement	
Resolution range (Å)	29.6–3.20
No. of reflections (working/free)	50 598/2705
No. of ColE9 residues	Chains A, B, D, and D — 488
No. of Im9 residues	Chains E and F — 83
	Chain G — 71
	Chain H — 68
No. of water molecules	23
*R*_work_^‡^/*R*_free_^§^ (%)	21.24/27.06
*B* average (Å^2^)	
Mean	93.4
ColE9	88.4
Im9	132.8
Water molecules	47.3
RMSD from ideal values	
Bond lengths (Å)	0.01
Bond angles (°)	1.3
Ramachandran^¶||^ (%) Favoured/acceptable/outlier	93.3/6.7/0.0

* Numbers given in brackets are from the last resolution shell.

† *R*_sym_ = (Σ*_hkl_*Σ*_i_*|*I_i_*(*hkl*)  − 〈*I*(*hkl*)〉)/Σ*_hkl_*Σ*I_i_*(*hkl*), where *I_i_*(*hkl*) is the intensity of the *i*th measurement of reflection (*hkl*) and 〈*I*(*hkl*)〉 is the average intensity.

‡ *R*_work_ = (Σ*_hkl_*|*F*_o_ − *F*_c_|)/Σ*_hkl_F*_o_, where *F*_o_ and *F*_c_ are the observed and calculated structure factors, respectively.

§ *R*_free_ is calculated as for *R*_work_ but from a randomly selected subset of the data (5%), which were excluded from the refinement.

|| Ramachandran analysis was carried out using the program MolProbity [48].

**Figure 3.**
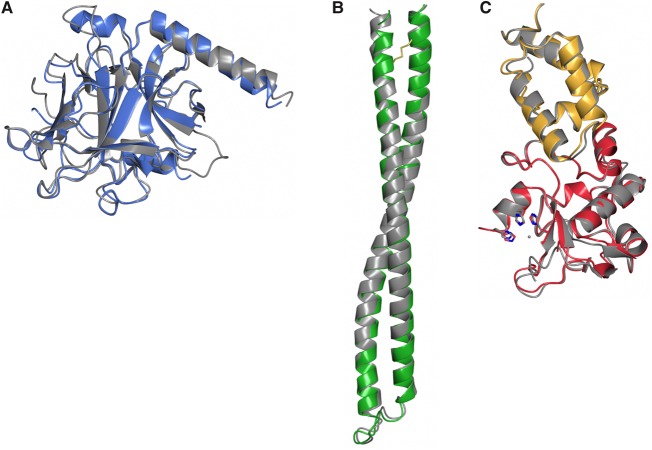
Structural superposition of three domains of ColE9 (shown in the same colour scheme as in Figure 2) with previously determined colicin structures (shown in grey). (**A**) T-domain and (**B**) R-domain superposed with ColE3 (2B5U, chain A); C_α_ RMSD = 1.16 and 1.16 Å for T- and R-domains, respectively. (**C**) The DNase–Im9 complex (1EMV); C_α_ RMSD = 0.74 Å.

As with other colicins (ColE3 and the pore-forming toxin ColIa), ColE9 adopts an extended structure with overall dimensions of 130 × 75 × 35 Å. The toxin consists of three structural domains. Residues 85–316 comprise the N-terminal T-domain, which is composed predominantly of β-sheet, with intervening loops and a single 23 residue α-helix (Figure 3A). Structural superposition of the ColE9 T-domain with that of ColE3 and ColE7 shows average root-mean-squared deviations (RMSD) of 1.25 Å (223 C_α_ atoms) and 1.16 Å (219 C_α_ atoms), respectively. ColE9 residues 317–452 form the 100 Å long coiled-coil R-domain of the colicin (Figure 3B). R-domains of ColE3 and ColE9 superpose with an RMSD of 1.16 Å (136 C_α_ atoms). The high structural similarity of the R-domain emphasizes that the disulphide used to constrain the R-domain in ColE9 has not introduced any distortion into the structure, which is reinforced by the observation that the thermodynamics of BtuB receptor-binding for the disulphided colicin are essentially identical with the wild-type protein [25]. The C-terminal DNase domain encompasses residues 453–580 of ColE9, which is in complex with its cognate Im protein, Im9 (Figure 3C). Structural superposition of the isolated E9 DNase domain–Im9 complex [29] shows a high degree of structural similarity to that of the intact ColE9–Im9 complex (0.74 Å), suggesting that crystal packing forces have not unduly distorted the complex (Supplementary Figure S1). The HNH motif of colicin DNases binds divalent metal ions that can often associate adventitiously during purification [30]. In the present structure, however, no additional electron density was observed in the HNH motif, suggesting that no metal ion is bound within the catalytic centre. This conclusion is further supported by the conformation of the four histidine residues, His551, His550, His575, and His579, all of which adopt conformations similar to those described previously for the E9 DNase–Im9 complex where a water molecule takes the place of the metal ion [31].

### The conserved colicin structural scaffold displays nuclease domains differently

**Figure 4.**
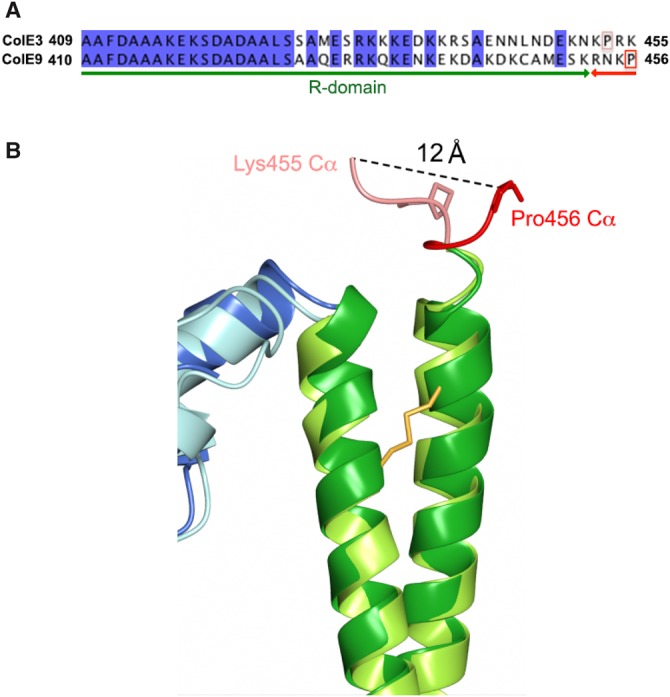
Structural differences at the boundary of R- and C-domains in ColE3 and ColE9. (**A**) Structure-based sequence alignment of ColE3 and ColE9. (**B**) Superposition of ColE3 and ColE9 structures at R- and C-domain boundaries. Distance between C_α_ atoms of equivalent residues in ColE3 (Lys455) and ColE9 (Pro456) is shown as a dashed line.

**Figure 5.**
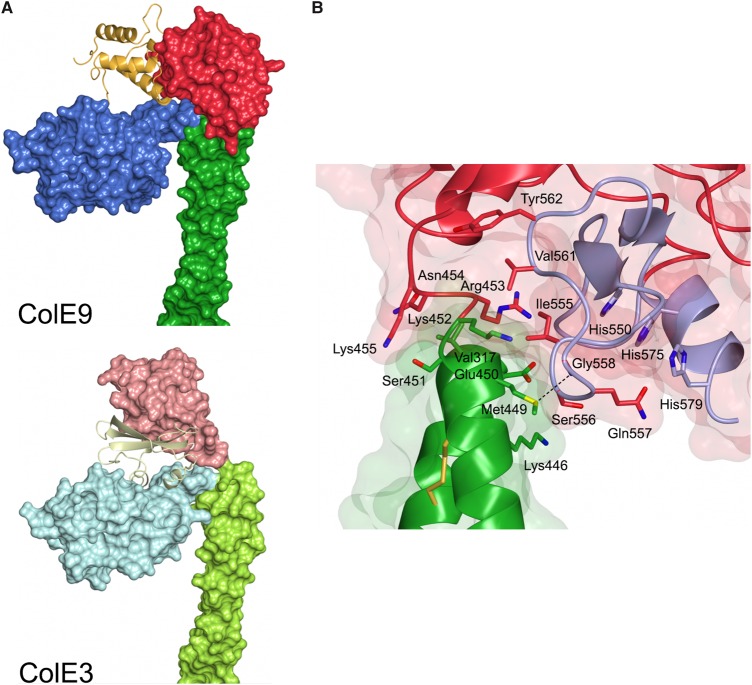
Im proteins Im3 and Im9 in complex with ColE3 and ColE9, respectively. (**A**) Im protein Im9 in complex with ColE9 interacts solely with the DNase domain (top panel), whereas in complex with ColE3 it is sandwiched between the DNase and translocation domains (bottom panel). (**B**) Interface between the DNase and R-domains in ColE9. HNH motif and active site histidine residues are shown in light purple.

E-type colicins use the same conserved structural scaffold of T- and R-domains to deliver three types of nuclease to the *E. coli* cytoplasm: ColE2, ColE7, ColE8, and ColE9 are DNases; ColE3, ColE4, and ColE6 are rRNases; and ColE5 is a tRNase. This functional diversity could be explained by each nuclease simply being attached to the conserved scaffold through a flexible linker, with the scaffold playing essentially no role in presentation of the nuclease to the cell once the colicin has bound its receptor, BtuB, through its R-domain. However, comparison of the ColE3 and ColE9 structures shows that, in fact, the scaffold plays an important role in how each nuclease is presented, which in turn influences how the associated Im proteins are accommodated within their respective complexes. The origin of these differences is the low level of sequence identity between the colicins at the C-termini of their R-domains, which are otherwise identical, especially residues 450–456 (Figure 4A). The C-terminal arm of the coiled-coil R-domain in both colicins ends at a glutamic acid (residue 450 in ColE9 and 449 in ColE3). In ColE3, this is followed by six residues (Lys450–Lys455), which adopt an extended conformation and end just prior to the first α-helix of the rRNase domain (Figure 4B). In contrast, the sequence linking the DNase to the R-domain in ColE9 is composed of one turn of a 3_10_ helix (Ser451–Asn454) followed by Lys455 and terminating at proline (Pro456) prior to the DNase domain. The result of these differences is that the DNase domain of ColE9 is rotated ∼90° away from the T-domain relative to ColE3, which is emphasized by the 12 Å movement of equivalent C_α_ atoms in the structural superposition of the two colicins (Figure 4B). The consequences of these structural rearrangements are three-fold. First, the orientation of Im3 in the ColE3–Im3 complex protein allows the Im protein to dock into a cleft on the T-domain with an interface area of 775 Å^2^ (Figure 5A). These contacts account for the higher affinity of Im3 for ColE3 relative to the isolated E3 rRNase [23]. Second, Im9 is rotated away from the T-domain of ColE9 leaving the cleft on the T-domain solvent accessible. The lack of an interaction with the T-domain explains why the affinity of Im9 for ColE9 and the isolated E9 DNase are identical [22]. Third, the DNase domain of ColE9 instead makes a hydrophobic contact with the R-domain (Val317, Lys446, and Met449–Lys452) mostly via an extended loop that links β-strands of the HNH motif of the nuclease (residues 555–558 and 561–562; Figure 5B). Since the isolated E3 rRNase domain binds Im3 more weakly than does the E9 DNase domain binds Im9, we speculate that the additional stabilization imparted by the T-domain might be needed to prevent the premature dissociation of Im3 from ColE3 in the producing organism, whereas this is not required for ColE9-producing *E. coli* cells.

### Nuclease colicins and pyocins share structural and topological similarities

One of the striking features of colicins is how varied the structures of these multidomain proteins can be even though they have a conserved domain arrangement (T–R–C) [7]. While some of the earliest colicin structures reported (ColIa and ColE3) showed the molecules to be highly extended, the majority of subsequent studies highlighted more compact protein architectures (ColN, ColM, ColB, and ColS4), emphasizing that an elongated shape is not a prerequisite for colicin action. Indeed, the only structural information on pyocins (L1 and M) shows them to be small compact molecules [32,33].

**Figure 6.**
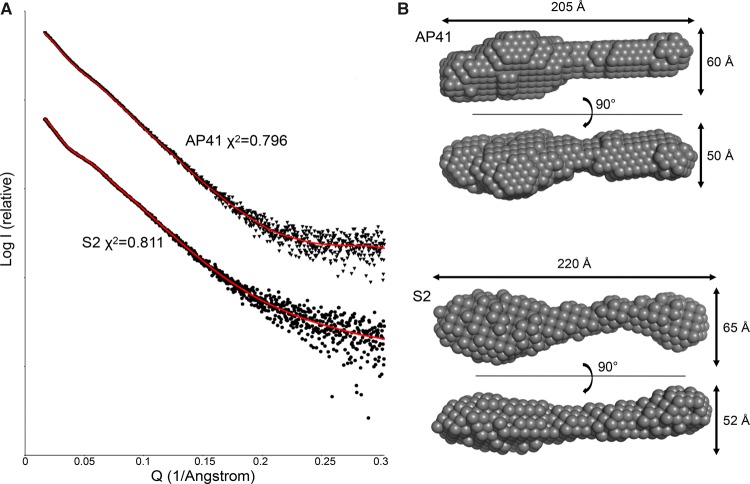
SAXS analysis of pyocins S2 and AP41 bound to their respective immunity proteins. (**A**) SAXS of the pyocins AP41–ImAp41 (triangles) and S2–ImS2 (circles) with the fit of the *ab initio* DAMMIF model (red line) and the error of the fit shown. (**B**) Filtered dummy atom models generated using DAMMIF for pyocin S2–ImS2 and AP41–ImAP41, which show their elongated extended conformations.

There is currently little structural information on intact nuclease S-type pyocins beyond their sharing similar C-terminal nuclease domains with colicins. Moreover, the order of T- and R-domains within these pyocins (R–T–C) is reversed relative to colicins (T–R–C), which may have implications for their mode of cellular import [6,19]. We therefore performed small-angle X-ray scattering (SAXS) experiments on the pyocin S2–ImS2 and AP41–ImAP41 complexes to ascertain the overall topology of these toxins (Figure 6). *Ab initio* models of the two pyocin–Im protein complexes were built using DAMMIF and averaged using DAMAVER and final *ab initio* models generated with DAMFILT fit well to the experimental scattering data (*χ*^2^ = 0.811 and 0.796 for S2–ImS2 and AP41–ImAP41, respectively). Molecular envelopes for both pyocins are highly elongated with a maximum particle dimension (*D*_max_) of 220 Å for pyocin S2–Im2 and 205 Å for pyocin AP41–ImAP41 (Figure 6). The molecular envelopes of both pyocins possess a large distinct globular region located at one end of the molecule, whereas the other end has a more slender appearance. This overall shape is strikingly different from the shape of ColE9 (Supplementary Figure S2) which, while having an extended helical R-domain, is 70 Å shorter than the pyocins.

**Figure 7.**
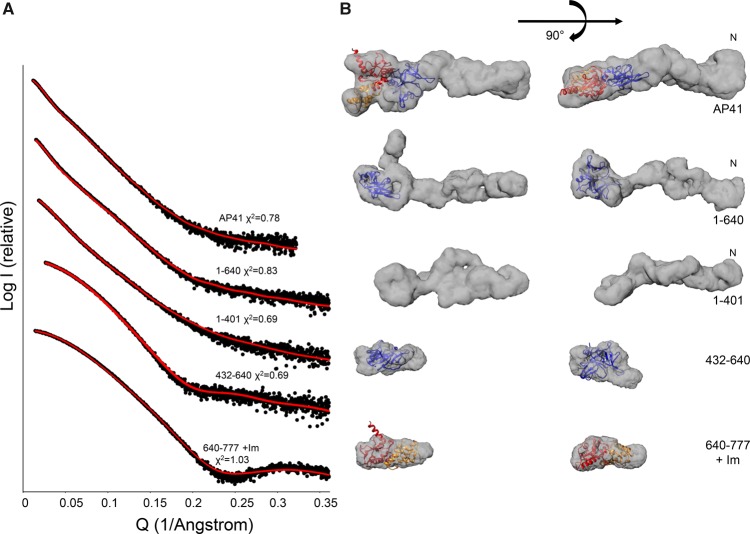
SAXS analysis of truncated pyocin AP41 constructs. (**A**) SAXS of pyocin AP41 fragments (closed circles) with the fit of the GASBOR model (red line) and the error of the fit shown. (**B**) Filtered dummy residue models generated using GASBOR for pyocin AP41 fragments. Cartoon representation of the AP41 DNase–Im complex shown in red and orange, respectively (PDBID: 4UHP) [20], and the S-type pyocin is shown in a blue cartoon representation (PDB ID: 3MFB) [34].

The low-resolution SAXS data do not allow identification of particular protein domains within the envelope. Therefore, in order to ascertain the domain positions, SAXS data were collected for a fragment series of AP41: the N-terminal region (residues 1–401), N-terminal and central regions (residues 1–640), central region (residues 432–640), and C-terminal DNase domain in complex with the cognate Im protein (AP41 residues 640–777 + Im; Figure 7). *Ab initio* models were generated which fit well to the experimental scattering data (0.69 < *χ*^2^ < 1.03). Repeat measurement of the full-length AP41–ImAP41 X-ray scattering was undertaken to ascertain any differences due to sample preparation, experimental set up, or data processing. There is a 10% difference in the *D*_max_ values, 205 and 185 Å, for the different measurements, respectively. The fit of the dummy atoms to this scattering curve is broadly similar to the earlier measurement (*χ*^2^ = 0.796 and 0.78, respectively); however, there is a pronounced kink and the envelope now looks more similar to that of S2–Im2 (Figure 6).

The pyocin AP41 DNase domain–ImAP41 complex envelope is compact with a slight elongation towards one side, maximum *D*_max_ = 222 Å. The crystal structure of the AP41 DNase domain–ImAP41 complex (PDB ID: 4UHP) [20] was manually fitted within this envelope using Chimera and the fit was refined within the Situs package (Figure 7). The correlation coefficient for the AP41 DNase domain–Im complex within the SAXS derived envelope is 0.87 (Table 2), and the domain fits well within scattering envelope. Similarly, the central region of pyocin AP41 (residues 432–640) can be described by a small, compact domain that is similar in sequence to the small mixed α/β-domain from a pectocin produced by *Pectobacterium atrosepticum* (PDB ID: 3MFB) [34] (Figure 7). The correlation coefficient of this pectocin domain to the SAXS data after refinement is 0.66, although in this instance the structure of the *P. atrosepticum* protein accounts for only 75% of the pyocin sequence. The protein fragment that encompasses the N-terminal half of the protein (residues 1–401), which is implicated in receptor-binding [19], has a greatly extended conformation that is ∼80% the length (162 Å) of the full-length protein, even though it accounts for only 50% of the protein sequence (Figure 7). No crystallographic information is available for this region of the pyocin but, as with ColE9, is predicted to contain a coiled-coil, which is consistent with its elongated structure.

**Table 2:** SAXS data fitting

	GASBOR *χ*^2^	Situs fit	Arbitrary fit
AP41–ImAP41	0.78	0.58	0.37
AP41 1–640	0.83	0.49	0.39
AP41 1–401	0.69	–	–
AP41 432–630	0.69	0.66	–
AP41 640–777 + ImAP41	1.03	0.87	–

The scattering data from the three different components — the N-terminal 1–401 fragment, the central pyocin S-type domain, and the C-terminal DNase–Im complex — allow the identification of these domains within the scattering envelopes of larger pyocin AP41 constructs. The scattering envelope of the full-length pyocin AP41–ImAP41 complex (Figure 7, top) contains an elongated thinner half and a larger wider half. The pyocin S-type domain of AP41 is separated from its DNase domain by a very short linker (five amino acids). These two domains fit well into the larger globular region of the molecular envelope and we propose that the elongated section could accommodate the N-terminal half of the protein (Figure 7), which is consistent with the elongated molecular envelope of AP41 1–401. The molecular envelope of AP41 1–640 is similar in length to the intact protein, suggesting that the DNase domain–Im protein complex contributes to the bulky protrusion, which in turn implies that this is the C-terminal end of the protein (Figure 7). As the pyocins AP41 and S2 have a high degree of sequence similarity [19] and similar overall molecular envelopes (Figure 6), we propose that the domains within pyocin S2 are similarly arranged. We validated the fits of the AP41 DNase domain–Im and S-type pyocin domains to the AP41 molecular envelope and obtained correlation coefficients of 0.58 and 0.49 for full-length AP41–Im and AP41 1–640, respectively. However, these correspond to only 45% of the whole protein (Table 2). To further validate these fits, the domains were placed arbitrarily within the molecular envelopes which gave poorer correlation coefficients of 0.39 and 0.37, respectively (Table 2).

## Conclusions

Protein bacteriocins are narrow-spectrum, species-specific antibiotics by virtue of discrete domains that bind receptor and translocator proteins within the cell envelope of their target species. Our structure for the ColE9–Im9 complex, only the second intact colicin nuclease structure after the ColE3–Im3 complex [21], shows how the conserved scaffold of T- and R-domains presents the E9 DNase–Im9 complex in a different orientation to that of the E3 rRNase–Im3 complex where the Im protein is sandwiched between the T- and C-domains. Since Im proteins are dissociated at the cell surface through an energy-dependent process [16], these differences are unlikely to influence the mechanism of import. The differential binding may, however, be related to the protection afforded to colicinogenic bacteria by Im proteins; Im3 binding to the isolated nuclease domain is weaker than Im9 where there is no difference in binding between the intact toxin and isolated nuclease domain [22].

Our data demonstrate for the first time that E-type nuclease colicins (E2–E9) and S-type pyocins (S2 and AP41) share a highly elongated structure, even though they share no sequence similarity beyond their nuclease domain (where this is of the same cytotoxic class). This is an intriguing observation given that the R-domain is purported to be N-terminal to the T-domain in pyocins, the opposite to the domain arrangement of colicins, which may signify differences in their import mechanisms. The other main structural difference to colicins is the apparent absence in S-type pyocins of an IUTD at the N-terminus of the toxin, which in colicins plays an important role in the translocation process. The IUTD is an 83-residue sequence at the N-terminus of E-type colicins that contains three protein–protein interaction-binding epitopes, two for the porin OmpF and one for TolB [35]. Following initial binding of the central R-domain to BtuB, the IUTD threads through a neighbouring OmpF to capture periplasmic TolB and thus forms the outer membrane translocon, which triggers entry of the nuclease into the cell [36]. Co-location of different outer membrane proteins within large clustered islands in the outer membrane of *E. coli* cells probably expedites the formation of such translocons [37]. There then follows a conformational change centred around the colicins coiled-coil R-domain, which is blocked by the disulphide used in the present study on ColE9 [24]. Positioning of the pyocin R-domain at the N-terminus coupled with the lack of an extended IUTD raises many questions as to how (or indeed if) a receptor-bound pyocin recruits translocation proteins to initiate import into the cell. In the case of colicins E3 and Ia, recruitment of translocation proteins (OmpF and Cir, respectively) is expedited by a 45° tilt, relative to the membrane surface, of the colicin when bound to its receptor. The pyocin would necessitate such an interaction with the receptor in order for its centrally located T-domain to meet potential translocation proteins on the membrane surface. Further work will be needed to test such a mechanism.

## Materials and methods

### Protein expression and purification

DNA sequence encoding *P. aeruginosa* PyoS2–ImS2 and PyoAP41–ImAP41 complexes were each cloned in tandem into the expression vector pET21a (Novagen) with the Im protein containing a non-cleavable C-terminal His_6_-tag. Deletion constructs of PyoAP41–ImAP41 were created by mutating the AP41 DNA sequence to insert restriction enzyme sites to facilitate subcloning. ColE9–Im9 Y324C and L447C was expressed and purified as described previously [24]. Briefly, proteins were expressed in the *E. coli* strain BL21 (DE3) in LB at 37°C with expression induced by 1 mM IPTG for 3–5 h. Proteins were purified by metal affinity chromatography followed by size-exclusion chromatography gel filtration using Superdex 75 or Superdex 200 in a buffer containing 50 mM Tris pH 7.5, 300 mM NaCl for ColE9–Im9, or 50 mM Tris pH 7.5, 150 mM NaCl for AP41 samples, or in a buffer containing 50 mM Tris pH 7.5, 200 mM NaCl for PyoS2–ImS2. The purity of the fractions was confirmed by Coomassie-stained SDS–PAGE and fractions with >90% purity according to the analysis with SDS–PAGE pooled and stored at −20 °C.

### Stopped-flow measurements

Pre-equilibrium fluorescence measurements were made on an Applied Photophysics SX20 stopped-flow spectrometer, using an excitation wavelength of 280 nm with slit widths of 5 nm, monitoring changes in tryptophan emissions above 320 nm selected with a cut-off filter. The association of ColE9 with Im9 in phosphate-buffered saline (PBS), pH 7.4, was measured under second-order conditions, using equimolar concentrations of each protein (0.35 µM after mixing) at 25 °C as described previously [22]. Four thousand data points were collected over time courses of 0.2 and 2 s, averaging 10 data acquisitions before analysis. The initial fluorescence enhancement (100 ms) was fitted to

$$F=F_{0}+\Delta F[E{]}_{0}^{2}k_{1}t/(1+[E{]}_{0}k_{1}t)$$

where [*E*]_0_ is the initial concentration of ColE9 and Im9, *F* is the fluorescence at time *t*, *F*_0_ is the initial fluorescence, and Δ*F* is the total chance in fluorescence divided by protein concentration. The slower fluorescence quench (200 ms–2 s) was fitted to a single exponential equation.

### Analytical gel filtration

Analytical size-exclusion chromatography was used to quantify the release of Im9 from the Y324C, L447C ColE9–Im9 complex in the presence of excess E9 DNase domain. Y324C, L447C ColE9–Im9 (16.4 µM) was incubated with a six-fold molar excess of E9 DNase domain in PBS buffer, pH 7.4, in the presence of protease inhibitor cocktail (set III, EDTA free, Calbiochem). After various incubation times between 0 and 156 h, aliquots of the sample were analyzed on a Superdex 200 10/300 GL column (GE) equilibrated in PBS, pH 7.4, allowing resolution of three peaks corresponding to ColE9/ColE9–Im9, E9 DNase·Im9, and free E9 DNase. The area of the E9 DNase·Im9 peak was integrated to quantify the amount of Im9 released as a function of time, with data fitted to a single exponential equation to determine the dissociation rate constant.

### Crystallization and structure determination

Crystallization trials with ColE9–Im9 complex were performed at various concentrations (10–140 mg/ml) using hanging drop and sitting drop vapour diffusion methods. The complex is highly soluble and has a tendency to form clusters of thin crystals. Best results were achieved using microseeding at a concentration of the complex of 90 mg/ml in 1.3 M sodium malonate pH 4.0, 18% polyethylene glycol 3350. A single crystal from the crystallization drop was directly transferred into liquid nitrogen. The single wavelength X-ray diffraction data were collected at 100 K on the Diamond Light Source beamline I02 using a Pilatus 6M detector. Data were measured with crystal-to-detector distance of 579.2 mm, using an oscillation range of 0.2°. Nine hundred images were collected to a maximum resolution of 3.2 Å. Recorded data were processed with XDS [38] and the reflection intensities were processed with COMBAT and scaled with AIMLESS [39] from the CCP4 program suite [40]. ColE9(Y324C;L447C)–Im9 crystal belonged to monoclinic space group *P*12_1_1 with unit cell dimensions *a* = 94.89, *b* = 116.05, and *c* = 150.37 Å. Matthews coefficient [41] indicated the presence of either five or four molecules in the asymmetric unit at 46 and 57% solvent content, respectively. The structure was determined by the molecular replacement method using the program PHASER [42]. The co-ordinates of the ColE3 T- and R-domains (PDB ID: 1JCH) and ColE9 DNase domain–Im9 complex (2GZG) served as a search model ensemble. Refinement was carried out using the program REFMAC5 [43]. The structure was visualized and rebuilt into the electron density using the program Coot [44]. The stereochemistry of the model was evaluated using the program MolProbity [45]. Data collection and refinement statistics are summarized in Table 1. Atomic co-ordinates and structural amplitudes have been deposited with the Protein Data Bank (PDB ID: 5EW5). For structure homology analysis, the server DALI [46] was used. To analyze the crystal packing, the PISA server at the European Bioinformatics Institute was used [47].

### Small-angle X-ray scattering data

Small-angle scattering data were collected at beamline B21 at Diamond Light Source. For each purified protein, construct data were collected for at least four different protein concentrations. Where there was evidence of low-angle, interparticle artefacts, datasets were merged to combine the low-angle region of low concentration samples with high-angle data for high concentration samples. SAXS data were processed with both the Scatter and ATSAS software suites [48,49]. Guinier approximation analysis and *P*(*r*) distribution were determined using Scatter software [48] (see Supplementary Figures S3 and S4). Dummy atom fitting was performed using multiple parallel runs of DAMMIF [50], which were filtered and averaged using DAMAVER [51]. These models were compared with dummy atom models generated by GASBOR to cross-validate the models [52]. The structures of the AP41 DNase–Im complex (PDB ID: 4UHP) [20] and the S-type pyocin domain (PDB ID: 3MFB) [34] were manually fitted into the molecular envelope using Chimera [53] and refined using Situs [54]. To further validate these fits, the domains were placed centrally within the molecular envelopes. In fitting the full-length protein, an additional constraint was imposed as all domains fitted within the envelope must be close in space. The positions were refined within SITUS and the obtained correlation coefficients were poorer at 0.39 and 0.37, respectively (Table 2). The SAXS envelope of ColE9 was calculated by simulating SAXS of ColE9 chains A and E using CRYSOL [55] and processing the scattering data as above.

## Abbreviations

CDI, contact-dependent inhibition; C-domain, cytotoxic domain; ColE3, colicin E3; ColE9, colicin E9; DNase, endonuclease; Im, immunity; Im3, immunity protein 3; Im9, immunity protein 9; IUTD, intrinsically unstructured translocation domain; PBS, phosphate-buffered saline; pmf, proton motive force; pyoAP41, pyocin AP41; pyoS2, pyocin S2; R-domain, receptor-binding domain; rRNase, ribosome-specific RNase; RMSD, root-mean-squared deviations; SAXS, small-angle X-ray scattering; T-domain, translocation domain; tRNase, tRNA-specific RNase.

## Author Contribution

A.K., J.A.W., A.J., I.J., L.C.M., N.G.H., and R.K. prepared samples and performed experiments. D.W. and C.K. devised the project. O.B., D.W., and C.K. gained funding for and oversaw the project. J.A.W., A.J., and C.K. prepared the first draft of the manuscript. All authors reviewed and contributed to the manuscript.

## Funding

This work was funded by a Biotechnology and Biological Sciences Research Council LOLA grant to C.K. [BB/G020671/1]. I.J. and L.C.M. were supported by studentships from the Wellcome Trust [093592/Z/10/Z] and [106064/Z/14/2], respectively.
